# T_1ρ_ magnetic resonance imaging quantification of early articular cartilage degeneration in a rabbit model

**DOI:** 10.1186/s12891-015-0810-0

**Published:** 2015-11-19

**Authors:** Si Shen, Hao Wang, Jing Zhang, Fei Wang, Meng Chen

**Affiliations:** Medical Imaging Center, The First Affiliated Hospital of Jinan University, Guangzhou, 510630 China; Pain Clinic, The First Affiliated Hospital of Jinan University, Guangzhou, 510630 China

**Keywords:** T_1ρ_, Magnetic resonance imaging, Early cartilage degeneration, Proteoglycan, Histology

## Abstract

**Background:**

Osteoarthritis (OA) is a serious problem in the recent aging society, and early diagnosis and intervention of articular cartilage degeneration are very important for the onset of OA. Therefore, development of newer MRI techniques is necessary and expected for detection of early articular cartilage degeneration.

**Methods:**

24 rabbits were randomly divided into four equal experimental groups (Group A, B, C, D) to establish articular cartilage models in different grades of early degeneration by injecting papain into the left knee joint cavity. Another 8 rabbits were considered as blank control (Group E), and then randomized into four subgroups (E_A_, E_B_, E_C_, E_D_). T_1ρ_ and T_2_-weighted images of the bilateral knee joints were obtained for rabbits by using 3.0 T MRI. Group A, B, C, and D were imaged respectively at 1, 2, 3, and 4 weeks post-operation, and E_A_, E_B_, E_C_, E_D_ underwent the same period imaging. Rabbits were sacrificed after scanning and the femoral condyle cartilage (FCC) was histological examined. T_1ρ_ values of the femoral condyle cartilage were measured and statistically analyzed, and contrasted with the histologic results.

**Results:**

T_1ρ_ values of the left side in experimental groups were significantly higher than the right side (*P* < 0.05), and which increased gradually with the passage of post-operation time (*P* < 0.05). Histological examination demonstrated the proteoglycan content of the left side decreased, and indicated the occurrence of early degeneration.

**Conclusions:**

T_1ρ_ MRI can sensitively and quantitatively reflect the change in proteoglycans prior to the morphologic alterations of articular cartilage, and T_1ρ_ value is gradually increased with a decrease in proteoglycan content, therefore that T_1ρ_ could potentially act as a reliable tool to identify early cartilage degeneration.

## Background

Osteoarthritis (OA) is the most common joint disorder worldwide, which is the major cause of mobility impaired and disabled in humans [1, 2]. It affects approximately 75 % of the population over 70 years of age, while a quarter of people aged over 55 have an episode of persistent knee pain, and there is a rapid increase in the number of the cases due to the aging of the population and the obesity epidemic [2–5]. This suggests that OA will continue to be a large and growing public health problem in the future [5, 6].

The most recent accomplishments have indicated that articular cartilage degeneration is highly likely to play a key role in the pathogenesis of OA, and early diagnosis and intervention of cartilage degeneration before the onset of irreversible changes, are very important for the prevention and treatment of OA [2, 7, 8]. For this purpose, considerable efforts have recently been in search of non-invasive techniques to sensitively and accurately assess the prophase of cartilage degeneration [9]. Articular cartilage starts early to degenerate from extracellular matrix (ECM) metabolism imbalance, which mainly involves a gradual loss of proteoglycan (PG), collagen damage and increased water content, and these macromolecular (biochemical) changes are not accompanied by significant structural disturbances in the tissue [10–13]. Among them, decreased PG content is an initiating factor and an important marker of early cartilage degeneration. Presently, it is believed that cartilage destruction results from large PG aggrecan degradation, which precedes the damage to collagen fibrillar network and is responsible for the whole changes [12–15]. In short, early cartilage changes are characterized by PGs depletion, and detection of PG content can identify early cartilage degeneration. Although conventional magnetic resonance imaging (MRI) could provide excellent detection of morphological changes in the thickness and structure of articular cartilage, it has some difficulties in discriminating minimal changes associated with the early stage of degeneration before morphological or clinical alterations [2, 12, 13]. Given as such, newer MRI techniques have emerged for investigation of the physiology and biochemistry of cartilage.

T_1ρ_ MRI is one of the most promising techniques focused on PG quantification [2, 12, 16]. The longitudinal relaxation time that occurs during the application of T_1ρ_ imaging is referred to as spin–lattice relaxation in the rotating frame, or T_1ρ_ relaxation time. T_1ρ_ relaxation time (T_1ρ_ value) can be measured to describe the slow-motion interactions between the macromolecule protons and bulk water protons at high static fields in vivo, and therefore indirectly reflect PG content [17–20]. As a consequence, T_1ρ_ imaging is mainly used for the evaluation of tissues composed partly of slow-frequency macromolecules, such as brain, articular cartilage, and intervertebral disc [21–23]. In articular cartilage, several studies have reported that a decrease in PG content is the major factor in change of T_1ρ_ value, and which has been shown to detect cartilage changes [13, 23, 24]. However, T_1ρ_ is still available on a research basic, and further studies are required to evaluate its value in early degeneration, and optimize the scanning sequence to create a reliable quantitative scale [2, 25–27]. Therefore, we designed the present study to confirm the sensibility and reliablity of T_1ρ_ in quantification of early cartilage degeneration with histology.

## Methods

### Ethics statement

The experimental protocol was approved by the Animal Care and Use Committee of Jinan University, Guangzhou, China. All procedures performed in this study involving animals were in accordance with the revised Animals (Scientific Procedures) Act 1986 in the UK and Directive 2010/63/EU in Europe.

### Preliminary experiment

In order to investigate the feasibility of this study, including training operations and sample size estimation, two healthy New Zealand white rabbits were performed the preliminary experiment first. Sample size was determined by the calculation of the pre-test results and the relevant literatures [12, 28].

### Animal model

Thirty-two healthy New Zealand white rabbits (16 male and 16 female, with a mean age of 8 months and weighting from 2.0 to 3.0 kg) were used for the formal experiment. Rabbits were randomly numbered 1–32, and divided into 5 groups. Numbers 1 to 6 were included in Group A, 7 to 12 in Group B, 13 to 18 in Group C, 19 to 24 in Group D, and numbers 25 to 32 were included in Group E (among the 8 rabbits in Group E, 2 rabbits were randomly selected for each subgroup E_A_, E_B_, E_C_, and E_D_, which were considered as the blank controls of Group A, B, C, and D, respectively). The left knees of experimental groups (Group A, B, C, D) were served as the experimental side to establish articular cartilage models at different stages of early degeneration with papain, which is a known and validated mediator of inducing PG catabolism [28–31], and the right knees acted as the control side. On the 1st, 4th, and 7th day of the experiment, the twenty-four rabbits of experimental groups were administered an injection of 0.5 ml of 1.6 % papain solution into the joint cavity of the left knee by using a 16-gauge hypodermic needle, and a control injection of the same amount of solution without papain in the right knee. The rabbits were sedated via ear vein injection of Ketamine (10 mg/kg) and Midazolam (1 mg/kg) while being monitored by an anesthesiologist for the period of the surgical procedure. Postoperatively, the rabbits were housed in individual cages without restriction of joint movement.

### MRI Protocol

T_1ρ_ and T_2_-weighted images of the bilateral knee joints were obtained for 32 rabbits by using a clinical 3.0 T MRI scanner (GE discovery MR750; the First Affiliated Hospital of Jinan University, Guangzhou, China) and human 8ch knee array coil. Group A, B, C, and D were scanned in sequence at 1, 2, 3 and 4 weeks post-operation (from the last injection of papain) respectively, and rabbits in the subgroups (E_A_, E_B_, E_C_, and E_D_) underwent MRI in the same period for comparison. For the duration of the imaging session (approximately 15 mins), each rabbit was under general anesthesia with intravenous Ketamine (8 mg/kg) and Midazolam (0.8 mg/kg), and the protocol included 2 sequences: the first, conventional T_2_-weighted imaging (T_2_WI) for morphological evaluation were acquired using a fast spin-echo (FSE) imaging on sagittal view, with the following parameters: repetition time/echo time (TR/TE) = 2000 ms/85 ms, slice thickness/spacing (thn/spa) = 2 mm/0 mm, field of view (FOV) = 12 × 12 cm, acquisition matrix = 352 × 224, NEX = 2; the second MRI pulse sequence used for a series of T_1ρ_-weighted sagittal images was 3D Magnetization-Prepared Angle-Modulated Partitioned k-Space Spoiled Gradient Echo Snapshots (3D MAPSS) with a spin-lock pulse amplitude of 500 Hz, under the following parameters: time of spin-lock pulse (TSL) = 0, 10, 40, or 80 ms, TR/TE = 7.8 ms/3.7 ms, thn/spa = 12 mm/0 mm, FOV = 12 × 12 cm, matrix = 256 × 128, NEX = 1, scan time = 3 min 33 s, and the B1 of spin-lock radio frequency (RF) was 0.145 Gauss.

### Histology

Immediately after imaging, rabbits in each group were euthanized by intravenous pentobarbital (100 mg/kg), and both knees were harvested, formalin-fixed, and decalcified. Tissue blocks of 0.5 cm in thickness were harvested from the medial and lateral femoral condyle cartilage (MFCC and LFCC) on the sagittal plane, and made into paraffin-embedded tissue sections. Morphology of the chondrocytes was observed after hematoxylin and eosin (H&E) staining, and the PG content was determined by Safranin-O and Fast Green staining [12, 32].

### T_1ρ_ quantification

Sun Advantage Workstation 4.5 (GE) was used for the post-processing of T_1ρ_ images. With an exponential decay, T_1ρ_ maps were reconstructed on a pixel-by-pixel basis by fitting a linear regression of the image intensity data to the following equation:

$$ \mathbf{S}\ \left(\mathbf{T}\mathbf{S}\mathbf{L}\right) = {\mathbf{S}}_{\mathbf{0}} \times \mathbf{exp}\ \left(-\mathbf{T}\mathbf{S}\mathbf{L}/{\mathbf{T}}_{\mathbf{1}\boldsymbol{\uprho }}\right), $$

where S is the signal intensity on T_1ρ_ images with the given TSL.

On the intermediate layer of the T_1ρ_ sagittal image, three regions of interest (ROIs) were placed from anterior to posterior on the images of MFCC and LFCC for measurement of the T_1ρ_ values. Each ROI was drawn using a circular tool by the radiologist with more than 10 years of the professional experience of MRI, and in order to reduce the influence of partial volume effect on results, the ROIs were not allowed to be set on the adjacent tissue. Average values of the three ROIs were calculated and recorded as the T_1ρ_ values of MFCC and LFCC, which were statistically analyzed.

### Image interpretation

The interpretations of images acquired were determined by two radiologists with more than 10 years of experience, which blinded to all experimental information and the final histological diagnosis, and independently readed the T1ρ original and T2-weighted images of the 32 rabbits. Each observer performed the same interpretation twice, with a delay of two weeks, to assess intraobserver agreement. The image interpretation results were determined according to the agreement and disagreement between the two observers as follows: the same results and the final results after discussion.

### Statistical analysis

Interobserver and intraobserver reliability of image interpretation was evaluated with the interclass and intraclass correlation coefficient (ICC): poor (< 0.40), fair (0.40 – 0.59), good (0.60 – 0.74), and excellent (0.75 – 1.00). The mean ± standard deviation of measurements were calculated for the T_1ρ_ values of MFCC and LFCC. A normal distribution of our data was shown by using the normality test, and then the differences in the T_1_ρ values of LFCC and MFCC within experimental groups were compared using one-way analysis of variance (ANOVA). Comparison of the T_1_ρ values in the control side between Group E and experimental groups was conducted with independent sample *T*-test. A *P* value of < 0.05 was considered statistically significant. Statistical analyses were performed with SPSS statistics software package, version 10.0 (SPSS Inc., Chicago, Illinois, USA).

## Results

### Analysis of images

**Fig. 1:**
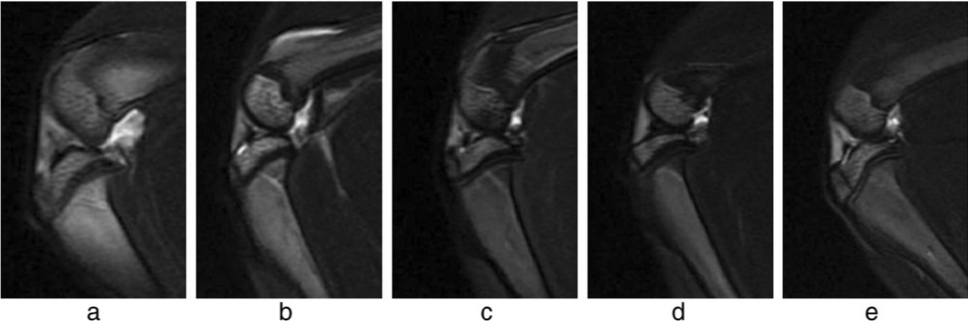
T_2_-weighted images of the FCC on experimental side (left side) in Group **e** and Group **a**-**d**. MRI with T_2_WI shown normal signal on sagittal view

**Fig. 2:**
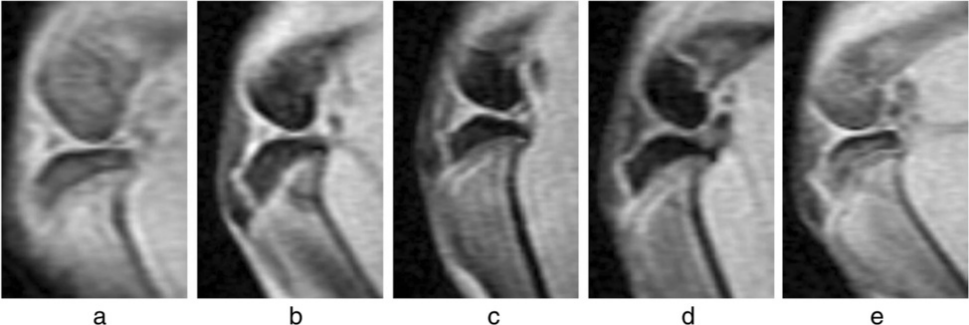
T_1_ρ original sagittal images of the FCC on experimental side (left side) in Group **e** and Group **a**-**d** before T_1ρ_ fitting. There was no significant imaging change of the cartilage

**Fig. 3:**
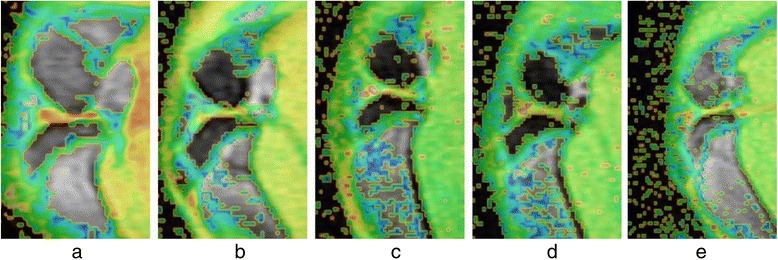
T_1_ρ pseudo-color images of the FCC on experimental side in Group E and Group **a**-**d** after T_1ρ_ fitting. Compared with Group E, the color of FCC in Group **a**-**d** gradually became darker, i.e., progressive changes from pale green to dark green were noted

**Fig. 4:**
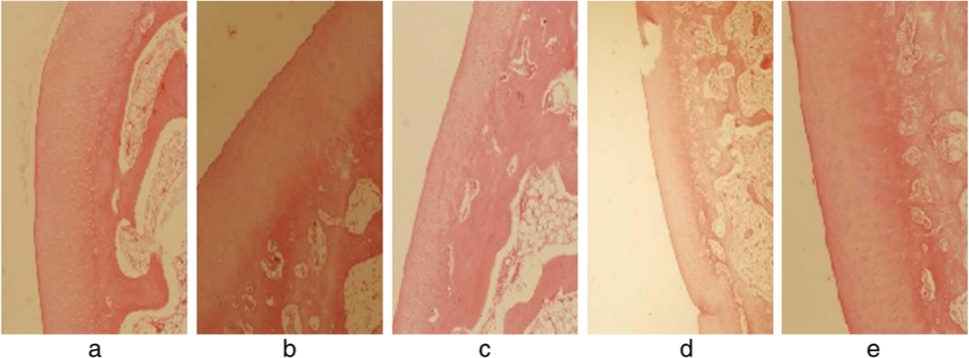
Shown were sections of H&E stained (×100) FCC from Group **e**, and the experimental sides of Group **a**-**d**. The normal morphology of chondrocytes in Group **a**-**d** gradually disappeared, and fibrosis of the ECM was observed, indicating early degeneration in the FCC of the experimental side

**Fig. 5:**
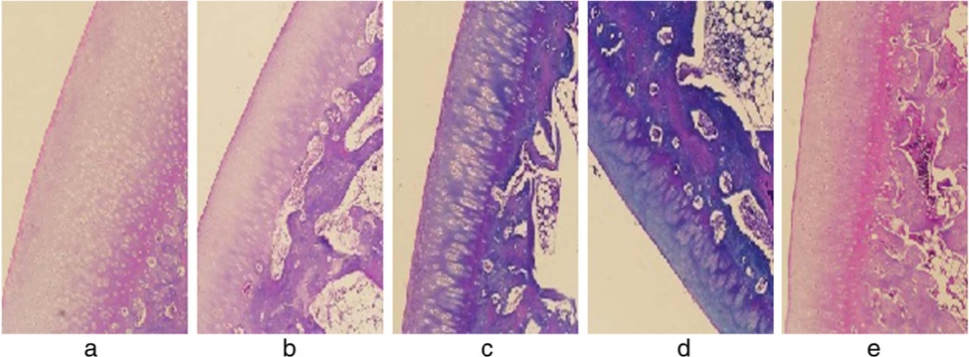
Shown were sections of Safranin-O and Fast Green stained (×100) FCC from Group **e**, and the experimental sides of Group **a**-**d**. In Group **a**-**d**, intensity of red positively correlated with the presence of glycosaminoglycans attached to PG gradually became pale, meanwhile the range of Fast Green staining of the collagen fibers gradually increased, indicating early cartilage degeneration

Two observers agreed perfectly that there was no imaging change of the femoral condyle cartilage (FCC) in the acquired T_1_ρ original and T_2_-weighted images of the 32 rabbits (Figures 1 and 2), in addition, there was no difference in images analysis between the first and the second interpretation by the same observer. Interclass and intraclass correlation coefficient were excellent, with *k* values of 1.00. T_1ρ_ pseudo-color images showed relatively significant chromatic aberration in the experimental sides compared with the control sides and Group E, and the color of the experimental sides were darker. Progressive changes from pale green to dark green were observed in the experimental sides of Group A-D (Fig. 3), which indicated the T_1ρ_ value increased gradually, and the process was consistent with the histological finding (Figs. 4 and 5).

### T_1ρ_ value differences of intra- and inter-group

**Fig 6:**
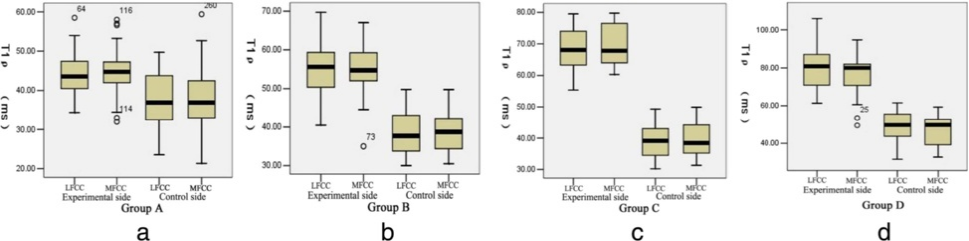
T_1_ρ values of LFCC and MFCC on the experimental side compared with the control side in Group **a**-**d**. T_1ρ_ values of the experimental side were significantly higher than the control side (*P* < 0.05)

**Fig. 7:**
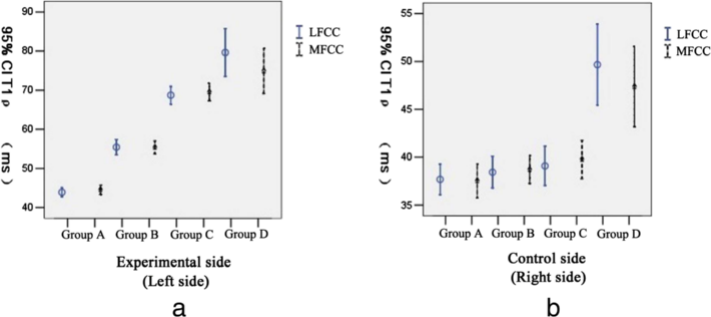
Differences in T_1_ρ values of LFCC and MFCC among Group **a**, **b**, **c**, and **d. 7a** From Group A to Group D, T_1ρ_ values of LFCC and MFCC on the experimental side increased gradually (*P* < 0.05). **7b**: No difference in T_1ρ_ values of the control side existed among Group A, B, and C (*P* > 0.05), while the T_1ρ_ value in Group D was obviously greater than in Group A, B, and C (*P* < 0.05)

T_1ρ_ values of LFCC and MFCC in the bilateral knees of rabbits are shown in Table **1**: T_1ρ_ values of LFCC and MFCC on the experimental side (left side) in Group A were 43.89 ± 4.87 (95 % CI, 42.75 – 45.04) and 44.53 ± 5.10 (95 % CI, 43.3 – 45.73), and T_1ρ_ values on the control side were 37.69 ± 6.73 (95 % CI, 36.11 – 39.28) and 37.53 ± 7.57 (95 % CI, 35.75 – 39.31), respectively; T_1ρ_ values of LFCC and MFCC on the experimental side in Group B were 55.41 ± 6.99 (95 % CI, 53.44 – 57.38) and 55.33 ± 5.98 (95 % CI, 53.64 – 57.02), and T_1ρ_ values on the control side were 38.45 ± 5.83 (95 % CI, 36.81 – 40.08) and 38.72 ± 5.14 (95 % CI, 37.27 – 40.16), respectively; T_1ρ_ values of LFCC and MFCC on the experimental side in Group C were 68.74 ± 6.40 (95 % CI, 66.47 – 71.01) and 69.54 ± 6.39 (95 % CI, 67.26 – 71.8), and T_1ρ_ values on the control side were 39.10 ± 5.77 (95 % CI, 37.05 – 41.15) and 39.76 ± 5.62 (95 % CI, 37.76 – 41.75), respectively; T_1ρ_ values of LFCC and MFCC on the experimental side in Group D were 79.63 ± 12.26 (95 % CI, 73.54 – 85.73) and 74.91 ± 11.6 (95 % CI, 69.14 – 80.68), and T_1ρ_ values on the control side were 49.67 ± 8.46 (95 % CI, 45.46 – 53.88) and 47.36 ± 8.44 (95 % CI, 43.16 – 51.56), respectively. In the experimental groups, the T_1ρ_ values of the experimental side were significantly higher than the control side (*P* < 0.05, Fig. 6). Fig. 7 showed that T_1ρ_ values of the experimental side increased gradually with the passage of post-operation time (*P* < 0.05), and there was no significant difference in T_1ρ_ values of the control side among Group A, B, and C (*P* > 0.05), while the T_1ρ_ values in Group D were obviously greater than in Group A, B, and C (*P* < 0.05). There was no difference in T_1ρ_ values on control side between Group A, B, C and the subgroup E_A_, E_B_, and E_C_ (*P* > 0.05, Table 1), while the T_1ρ_ values of LFCC and MFCC on the control side in Group D were obviously greater than in subgroup E_D_ (*P* < 0.05, Table 1) . There was no significant difference in T_1ρ_ values between the LFCC and MFCC, within the bilateral knees of Group A-D (*P* > 0.05, Table 1). Finally, No difference of T_1ρ_ values existed in Group E (*P* > 0.05).

**Table 1:** T_1ρ_ values of LFCC and MFCC in the bilateral knees of rabbits expressed as the mean ± standard deviation

T1ρ (ms)	Group A (1 week)		Group B (2 weeks)		Group C (3 weeks)		Group D (4 weeks)		Group E^e^ (Blank control)			
	Experimental side	Control side^a^	Experimental side	Control Side^b^	Experimental side	Control Side^c^	Experimental side	Control Side^d^	E_A_ right side^a^	E_B_ right side^b^	E_C_ right side^c^	E_D_ right side^d^
LFCC	43.89 ± 4.87	37.69 ± 6.73	55.41 ± 6.99	38.45 ± 5.83	68.74 ± 6.40	39.10 ± 5.77	79.63 ± 12.26	49.67 ± 8.46	39.11 ± 4.51	41.77 ± 8.05	39.38 ± 7.6	45.33 ± 12.42
95 % CI	42.75-45.04	36.11-39.28	53.44-57.38	36.81-40.08	66.47-71.01	37.05-41.15	73.54-85.73	45.46-53.88	36.79-41.44	37.31-46.23	33.53-45.23	14.48-76.18
MFCC	44.53 ± 5.10	37.53 ± 7.57	55.33 ± 5.98	38.72 ± 5.14	69.54 ± 6.39	39.76 ± 5.62	74.91 ± 11.6	47.36 ± 8.44	38.50 ± 6.48	40.77 ± 5.43	41.43 ± 6.42	35.08 ± 4.88
95 % CI	43.3-45.73	35.75-39.31	53.64-57.02	37.27-40.16	67.26-71.8	37.76-41.75	69.14-80.68	43.16-51.56	35.17-41.80	37.75-43.78	36.49-46.37	22.94-47.22

^*^There was no significant difference in T_1ρ_ values between the LFCC and MFCC, within the bilateral knees of Group A, B, C, and D (*P* > 0.05)

^a^Between the control side of Group A and the subgroup E_A_, no difference in T_1ρ_ values of LFCC existed (t = −0.98, *P =* 0.33), and also no difference in T_1ρ_ values of MFCC existed (t = −1.05, *P =* 0.30)

^b^Between the control side of Group B and the subgroup E_B_, no difference in T_1ρ_ values of LFCC existed (t = −1.12, *P =* 0.27), and also no difference in T_1ρ_ values of MFCC existed (t = −1.51, *P =* 0.14)

^c^Between the control side of Group C and the subgroup E_C_, no difference in T_1ρ_ values of LFCC existed (t = −1.51, *P =* 0.14), and also no difference in T_1ρ_ values of MFCC existed (t = −1.30, *P =*0.20)

^d^The T_1ρ_ value of LFCC on control side in Group D was significantly higher than in subgroup E_D_ (t =2.49, *P =* 0.02), and the T_1ρ_ value of MFCC was obviously greater than in subgroup E_D_ too (t = 4.70, *P =* 0.00)

^e^No difference of T_1ρ_ values existed in Group E (*P* > 0.05)

### Histodiagnosis

**Fig. 8:**
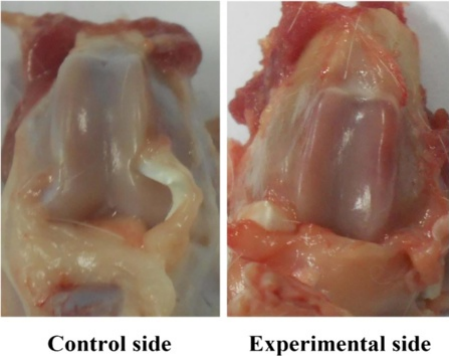
Gross specimens of a rabbit model in the experimental groups. The FCC of the bilateral knees was observed to be smooth and with little change in morphology

This study performed the injections of papain on rabbits to produce a decline in PG content for setting up the animal models of early articular cartilage degeneration, and the histodiagnosis substantiated the occurrence of biochemical changes in the FCC, which with little change in morphology. Figure 8 showed that the LFCC and MFCC of the specimens were smooth and devoid of morphologic alteration upon histological inspection. Figure 4 revealed early degenerative changes in FCC on the experimental side because of the gradually disappearing of normal chondrocytes and the fibrosis of ECM. And there was a visible reduction in histological staining of PG by Safranin-O for papain-treated FCC as compared with control FCC, and the loss of PG was proportional to the degree of cartilage degeneration, meanwhile the range of Fast Green staining of the collagen fibers gradually increased (Figs. 4 and 5). However, there were no significant change in the FCC on control side (except in Group D) and Group E; there was mild degeneration in the FCC on control side of Group D. Histological verification was determined by the observer with the professional experience of pathology.

## Discussion

In the present study, the primary purpose was to investigate whether T_1ρ_ MRI could be uesd as a means to detect biochemical changes prior to the morphologic alterations in articular cartilage with histology. Out of the conventional MRI methods, T_2_WI is commonly used to depict the morphological changes in cartilage, such as fibrillation, fissure, and partial- or full-thickness defect, which are preceded by biochemical changes of ECM [12, 33, 34]. Even though it can clearly show morphological abnormalities of cartilage, and has relatively high detection rates for the late cartilage degeneration, which mostly indicate the progressed stage of OA with irreversible variation of the cartilage, it was limited in detecting ultrastructural changes associated with early cartilage degeneration [2, 35, 36]. In this study, T_2_-weighted images were used to assess whether the morphologic alterations had occurred in the FCC of rabbit models or not, and were taken as a representative of conventional MRI to contrast with T1ρ images on the capability for characterizing the micro-changes in the prophase of cartilage degeneration. The histological examination demonstrated that early cartilage degeneration had already existed in the experimental sides of Group A-D, however, T_2_WI showed there was no obvious imaging change of the cartilage, and T_1ρ_ was highly sensitive to reflecting the biochemical micro-changes due to the early stages of FCC degeneration. It had suggested that T_1ρ_ MRI was a physiological imaging technique for detecting the biochemical state and micro information of tissue at the molecular level, and can identify early lesions which cannot be identified by routine MRI sequences.

By mean of an animal model, this study aimed to measure the T_1ρ_ value of FCC for quantitative reflection of PG depletion, which was the earliest finding of early cartilage degeneration. Toward this end, we successfully established a rabbit model of PG degradation inducing FCC degeneration. Histodiagnosis verified that the model highly replicated the pathological process of early articular cartilage degeneration, and

Group A, B, C, and D were at different stages of early degeneration, which ensured the reliability of the results. In our experiment, to select the rabbit as a model was for the reason that there was a high degree of similarity between the biochemical properties of articular cartilage in a rabbit and a human, such as the main composition of ECM, the morphology and alignment of chondrocytes, and the pathological mechanism of cartilage degeneration in rabbits also resembled human being [37, 38]. We chose to focus on the FCC mostly because that was readily identified by using surface coil for generating images, and easy to dissect from the knee joint thereby histological examination.

Histological sections showed that there was a visible reduction in staining intensity of PG in papain-treated cartilage as compared with control cartilage, and the red of staining in Group A-D gradually faded, while the normal morphology of chondrocytes gradually disappeared with the emergence of fibrosis in ECM over time. All of that indicated the degree of early cartilage degeneration progressively increased from Group A to Group D, and PG depletion was the characteristics of early cartilage degeneration, its loss was proportional to the degree of cartilage degeneration. With comprehensive analysis of T_1ρ_ imaging, a strong correlation was revealed between changes in PG content and T_1ρ_ value. The result of this study showed a obviously difference in T_1ρ_ values of FCC between experimental side and control side, the T_1ρ_ values on the experimental side were significantly higher than the control side. Moreover, there was a trend of gradual increase in T_1ρ_ values with the passage of post-operation time (after the last papain injection). This result was consistent with the loss of PG determined by the histodiagnosis, which suggested that a decreased PG content induced the increase of T_1ρ_ value, and the increased T_1ρ_ value reflected the decreased PG content. However, there was no significant difference in T_1ρ_ values between the control sides of Group A-C and the blank group (Group E), and histological examination showed that there was no cartilage degeneration in the control sides (except Group D) and Group E, which indicated the scientific validity of the case–control design and the objective presence of T_1ρ_ relaxation time extension in the experimental-side cartilage. Therefore, it can be clearly demonstrated based on the above analyses that the results of the present study were supported by the histological evidence, the increased T_1ρ_ value suggests early cartilage degeneration, and was positively correlated with the degree of cartilage degeneration. In addition, T_1ρ_ values increased in the control sides of Group D, and the phenomenon was consistent with the histological result. We speculated that this result was caused by the compensatory overload in the control-side cartilage due to cartilage damage of the experimental side, and such speculations surely warrants further investigations. Furthermore, we also found that 1 week after the last injection of papain, there was a significant difference in T_1ρ_ values of Group A between the experimental side and control side prior to cartilage degeneration shown by histological examination. This showed that the sensitivity of T_1ρ_ imaging may exceed that of histological examination for ultra-early low-level cartilage lesions, however, which should be verified by further research.

## Conclusions

In conclusion, the main contribution of this study lay in the investigation of T_1ρ_ imaging to quantitatively reflect biochemical changes in the initial phase of cartilage degeneration. The results suggested that T_1ρ_ value was a sensitive indicator for accurately detecting early articular cartilage degeneration, its increasing was closely related to the degenerative change. Therefore, T_1ρ_ MRI could be potentially used for the prospective and quantitative evaluation of early cartilage degeneration, and might allow intervention of cartilage degeneration at the earliest stages. However, T_1ρ_ technique was still available with only a few types of MR scanners (minimum usable field strength of 3.0 T) with specialized modified pulse sequences on a research basic, and it had not been widely applied [2, 25, 26]. The present study is also a preliminary exploration of the T_1ρ_ imaging in the evaluation of articular cartilage, and there were some difficulties with the T_1ρ_ technique, such as the repeatability of T_1ρ_ imaging, the setting of scanning parameters, the design of the T_1ρ_ image reconstruction program and the quality of the imaging. But despite all this, with the improvement of T_1ρ_ technique and optimization of the scanning sequence, T_1ρ_ MRI may be a useful tool for detecting early cartilage degeneration in clinical practice.

## Abbreviations

ECM: extracellular matrix
FCC: femoral condyle cartilage
FOV: field of view
LFCC: lateral femoral condyle cartilage
MFCC: medial femoral condyle cartilage
MRI: Magnetic resonance imaging
OA: Osteoarthritis
PG: proteoglycan
ROI: region of interest
T_2_WI: T_2_-weighted imaging
TE: echo time
TR: repetition time
TSL: time of spin-lock pulse
